# Surface Chemistry Aspects of Ion Exchange in Basic Copper Salts

**DOI:** 10.3390/molecules30010021

**Published:** 2024-12-25

**Authors:** Sebastian Skupiński, Marta Kalbarczyk, Daniel Kamiński, Marek Kosmulski

**Affiliations:** 1 Laboratory of Electrochemistry, Lublin University of Technology, Nadbystrzycka 38, 20-618 Lublin, Poland; s.skupinski@pollub.pl (S.S.); m.kalbarczyk@pollub.pl (M.K.); 2 Institute of Chemical Sciences, Maria Curie-Skłodowska University, Maria Curie Skłodowska, Square 3, 20-031 Lublin, Poland; daniel.kaminski@mail.umcs.pl

**Keywords:** copper basic salts, electrophoresis, TGA, XRD, specific surface area, SEM

## Abstract

Brochantite was precipitated using stoichiometric amounts of CuSO_4_ and NaOH and characterized by scanning electron microscopy, specific surface area, thermogravimetric analysis, and zeta potential. Brochantite can be converted into paratacamite, basic copper bromide, and copper phthalate by shaking the powder with solutions containing excess corresponding anions. By contrast, attempts to convert brochantite into basic iodide, acetate, nitrate, or rhodanide in a similar way failed, that is, the powder after shaking with solutions containing excess corresponding anions still showed the powder X-ray diffraction pattern of brochantite. Successful ion exchange resulted in a decrease in the specific surface area by a factor of 10, but the specific surface area was unchanged when attempts to exchange the anion failed. Interestingly enough, paratacamite can also be converted into brochantite by shaking with solution containing excess sulfate. Brochantite and paratacamite obtained by precipitation and the salts obtained by ion exchange showed a negative zeta potential at pH > 9.

## 1. Introduction

Basic copper salts, especially brochantite Cu_4_(OH)_6_SO_4_ and atacamite Cu_2_Cl(OH)_3_, occur as natural minerals [1,2]. They are also common corrosion products on copper and its alloys [3,4]. The corrosion of copper is important in electrical engineering, especially in outdoor applications, e.g., in power cables. Due to acid rain, basic copper salts are among the corrosion products, and their properties affect the corrosion process. The practical applications of basic copper salts include forage additives, especially for poultry, and fungicides in horticulture [5].

Basic copper salts are sparingly soluble in water, and they act as anion exchangers [6], that is, the anion can be exchanged for another anion when a basic copper salt is aged with a solution containing excess water soluble salt of another anion. Jiménez-López et al. [6] succeeded in reversible anion exchange between basic copper acetate and basic copper iodide, that is, acetate (obtained by precipitation) was converted into iodide, and iodide (obtained from acetate by ion exchange) was converted into acetate when soaked in solution containing excess corresponding anions. Basic copper salts other than acetate and iodide were not studied in [6]. In addition to X-ray diffraction (XRD) used to confirm the structure, Jiménez-López et al. used IR spectroscopy, diffuse reflectance spectroscopy, scanning electron microscopy (SEM), and extended X-ray absorption fine structure (EXAFS) spectroscopy to characterize their specimens.

Different aspects of ion exchange in basic copper salts have been extensively studied [7,8]. Stanimirova et al. [7] studied four different basic copper sulfates, three different basic copper chlorides, and one basic copper nitrate for possible conversion into other basic copper salts by ion exchange. They used XRD and SEM to characterize their specimens. Atacamite, paratacamite, and brochantite did not participate in ion exchange reactions. By contrast, basic copper nitrate could be easily converted into other basic copper salts by ion exchange.

In this study, we present the effects of anion exchange on the surface chemistry of basic copper salts, especially on their specific surface area and ζ potential. The ζ potential is an important property of fine particles in dispersions, for example, it governs the stability of dispersions against coagulation and sedimentation. Dispersions are stable when the absolute value of the ζ potential is high (typically > 50 mV), and they are unstable when the absolute value of the ζ potential is low (<10 mV). The ζ potential of many materials is pH dependent. Typically, the ζ potential is positive at low pH, and it is negative at high pH. The pH value where the ζ potential equals zero is termed the isoelectric point (IEP), and it is often reported as a part of material characterization. Moreover, the ζ potential governs other properties of solid–liquid interfaces and of fine particles, including the contact angle, adhesion, ability to adsorb ions, recovery by flotation in minerals engineering, etc. More details on the significance of the ζ potential and the IEP can be found elsewhere [5].

Moreover, characterization of brochantite and paratacamite obtained by precipitation (termed “original salts” as opposed to the salts obtained by ion exchange) and of the products of anion exchange by XRD, electron microscopy, and TGA is presented.

## 2. Materials and Methods

### 2.1. Reagents

The reagent grade chemicals were obtained from POCh (Lublin, Poland).

### 2.2. Preparation of Original Particles

The following reactions were performed in plastic containers to obtain paratacamite and brochantite, respectively:

2CuCl_2_ + 3NaOH → Cu_2_Cl(OH)_3_ + 3NaCl (1)

4CuSO_4_ + 6NaOH → Cu_4_(OH)_6_SO_4_ + 3Na_2_SO_4_ (2)

Fresh 1 M solution of Cu(II) salt was quickly poured into a stoichiometric amount of fresh 1 M solution of NaOH under vigorous stirring. The stirring was continued for 1 day at room temperature. The precipitate was washed with water. The purified precipitate was dried at 80 °C in plastic for 1 day and at 130 °C in glass for 1 day. The above recipes have been successfully used by the present authors and by others (see [5] and references therein) to obtain pure paratacamite and brochantite, respectively, from easily available reagents. Paratacamite and brochantite were selected as the polymorphs for ion exchange experiments, which show long-term stability in aqueous solutions over the temperature range studied in this paper, in contrast to other polymorphs of basic copper chloride and sulfate, respectively, which are less stable.

Moreover, the XRD patterns of pure paratacamite and brochantite are easily available, which is not always the case for other polymorphs of basic copper chloride and sulfate or for other basic copper salts. According to the RRUFF repository, the major peaks of paratacamite (ordered by intensity) are at 16.27, 39.87, 32.46, 53.7, 30.91, 32.87, 50.23, 18.96, 38.47, 44.5, and 47.89 degrees, and the major peaks of brochantite are at 22.78, 16.54, 13.88, 33.44, 35.62, 35.6, 22.76, 27.96, 30.58, 34.46, 52.51, 52.57, 28.01, 17.58, 61.55, 36.35, 36.58, 36.5, 43.52, and 11.33 degrees. These peaks are visible as separate peaks for single crystals, but several peaks overlap for fine powders.

### 2.3. Ion Exchange

The ion exchange experiments were carried out with brochantite from one lot in air-tight plastic containers. Typically, about 0.5 g of brochantite was shaken with 10 mL of solution containing about 1–2 g of water-soluble salt for 1 week. Then, the solid was separated by centrifugation and washed with water. The supernatants were collected and analyzed for sulfate concentration. The solids were dried as described above for the initial materials. The conditions of anion exchange are summarized in Table 1.

The prefix in the code denotes the initial material: B = brochantite, P = paratacamite. The absence of remarks in Table 1 denotes standard conditions of anion exchange, that is, 1 week at room temperature. In a few specimens, the temperature was elevated to 50 °C. In one specimen (B015), the time of anion exchange was extended to 3 weeks (rather than 1 week). In one specimen (B001), the clear supernatant was removed after 1 week and replaced with fresh solution. Then, the usual procedure was repeated with fresh solution. In two specimens (B014 and P016), the clear supernatant was removed after 1 week and replaced with fresh solution. After another week, the clear supernatant was removed and replaced with fresh solution. Then, the usual procedure was repeated with fresh solution. The discussion of the specific surface areas presented in Table 1 can be found in Section 3.4.

### 2.4. Characterization of Particles

The initial materials (brochantite and paratacamite) were characterized by scanning electron microscopy by means of a Quanta 3D FEG from FEI (Hillsboro, OR, USA). The conditions of the measurements are reported in the bottom of the images. XRD was used to confirm the conversion of brochantite into another salt (Table 1). We were especially interested in the presence of brochantite peaks and of the peaks of other copper salts that might have been obtained by anion exchange. Empyrean equipment from PANalytical (Almelo, The Netherlands) was used to collect the XRD patterns. The intensity was measured in 0.013° steps, each for 148.92 s. The XRD patterns of our specimens were compared with the standard patterns available in the RRUFF database.

Analysis of the supernatant for sulfate was another method used to confirm anion exchange. The combined supernatants from each specimen were mixed with excess acidified Ba(NO_3_)_2_ solution, and the amount of exchanged sulfate was determined from the mass of precipitated BaSO_4_.

The above two methods indicated if ion exchange was successful. Moreover, several other analyses were used to characterize both the initial materials and the products of anion exchange.

Gemini V from Micromeritics (Norcross, GA, USA) was used to measure the adsorption of nitrogen at 77 K. The BET specific surface areas were calculated from 7 data points at *p*/*p*_0_ ranging from 0.05 to 0.3.

The electrophoretic mobility and particle size were measured by means of a Malvern Zetasizer at 25 °C with fresh dispersions of all specimens in 0.001 M NaCl at pH 5–11. The pH was adjusted by the addition of small volumes (0.05–0.5 mL per 25 mL of dispersion) of dilute solutions of HCl and NaOH. The volumes and concentrations of acid and base in particular dispersions were adjusted on a trial and error basis to have at least one data point every 1.5 pH units in each series, that is, to avoid broad “unexplored” pH ranges. The pH was measured directly before the electrokinetic measurement. Most electrokinetic measurements were performed in fresh dispersions, that is, dispersions prepared less than 2 h before the electrokinetic measurement. Three series of electrokinetic measurements were performed with the original brochantite. Two series refer to fresh dispersions of freshly precipitated brochantite on the one hand and with the same brochantite after 2 months of storage (marked as “repeated”) on the other. The third series refers to dispersions aged for 3 days before the electrokinetic measurement.

The zeta potential was calculated by means of the Smoluchowski equation. In all graphs showing the zeta potential, symbols represent the experimental data and curves represent the 4th degree polynomial fitted to the data points. Curves were added to guide the eye. There is no physical theory behind use of the 4th degree polynomial rather than any other fitting curve.

Selected specimens were also characterized by TGA analysis (Netzsch STA 449 F3 Jupiter) from Netzsch, Selb, Germany. Paratacamite was studied under air flow and brochantite was studied under nitrogen flow. The heating rate was 1 K/min (lower than in most similar studies).

## 3. Results and Discussion

### 3.1. Initial Particles

The recipes described in the Materials and Methods section resulted in precipitation of brochantite and paratacamite rather than other polymorphs of Cu_4_(OH)_6_SO_4_ and of Cu_2_Cl(OH)_3_, respectively. Different conditions of precipitation lead to different polymorphs of copper basic sulfate [9], but detailed studies of such effects are beyond the scope of the present paper.

**Figure 1:**
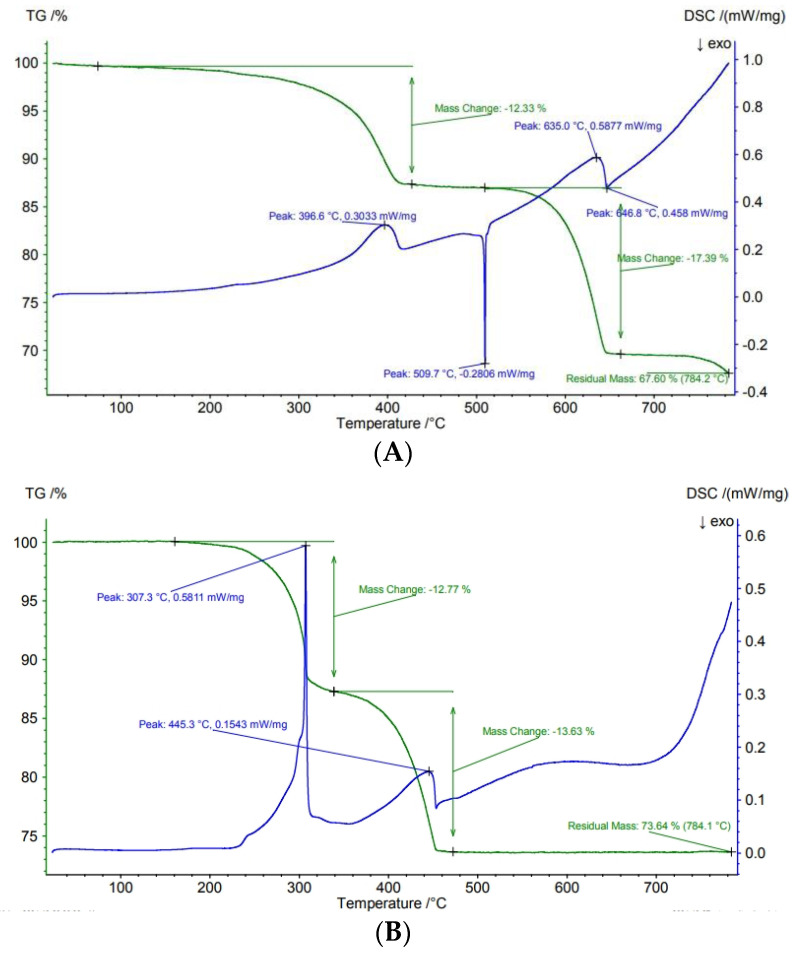
TGA curves of original brochantite (**A**) and paratacamite (**B**).

The results of the TGA analysis of original brochantite and paratacamite are presented in Figure 1. Brochantite lost 12.33% of its mass between 50 and 420 °C and 17.39% of its mass between 520 and 670 °C. These curves correspond to the loss of 3 molecules of water per one Cu_4_(OH)_6_SO_4_ unit in the first step (to produce 3CuO·CuSO_4_), and to the loss of 1 molecule of SO_3_ per one Cu_4_(OH)_6_SO_4_ unit in the second step (to produce CuO). The final loss of mass at >750 °C corresponds to the partial reduction of CuO to Cu_2_O under nitrogen flow. Kratohvil and Matijevic [10] reported a thermogravimetric curve of brochantite, but they did not report the experimental conditions. The final mass loss of 31.4% was reached at 750 °C. Tanaka et al. [11] studied several brochantites, but they only reported the numerical values of mass % of water (12.4 to 12.8) and of SO_3_ (17.3 to 17.9) obtained from their TGA curves rather than the complete curves.

Paratacamite was analyzed under air flow. It lost 12.77% of its mass between 50 and 350 °C and 13.63% of its mass between 350 and 460 °C. These curves correspond to the loss of 3 molecules of water per two Cu_2_Cl(OH)_3_ units in the first step (to produce 3 CuO·CuCl_2_), and to the replacement of 1 molecule of Cl_2_ with one oxygen atom per two Cu_2_Cl(OH)_3_ units in the second step (to produce CuO). The latter reaction is well known as a part of the Deacon process. The presence of oxygen in air prevented reduction of CuO to Cu_2_O. The thermogravimetric curves of paratacamite under nitrogen flow are more complicated because of an additional mass loss due to the reduction of CuO to Cu_2_O and to the evaporation of volatile copper chlorides. The later phenomenon is well known [12], and its investigation is beyond the scope of the present study.

Garcia-Martinez [13] obtained similar TGA curves of paratacamite (air flow and still air, 2 K/min) as those presented in Figure 1. Wang et al. [14] reported a thermogravimetric curve of their paratacamite (γ-Cu_2_(OH)_3_Cl), suggesting a much higher temperature of decomposition to CuO than that shown in Figure 1. They performed their experiments under argon flow with a heating rate of 10 K/min. Apparently, their heating rate was too high to complete the reaction at lower temperatures. Moreover, they observed the reduction of CuO to Cu_2_O, similar to that shown for brochantite in Figure 1. Ul Haq et al. [15] reported similar results as Wang et al. [14], although they performed their experiments under air flow with a heating rate of 10 K/min, and we interpret their results in a way similar to way described above for the results by Wang et al. [14].

Ul Haq et al. [15] interpreted their high mass loss in terms of octahydrate of paratacamite rather than anhydrous salt as the starting material. We disagree with such an interpretation because the XRD pattern of their specimen matches that of anhydrous paratacamite. Apparently, their high mass loss was due to evaporation of volatile copper chloride(s).

**Figure 2:**
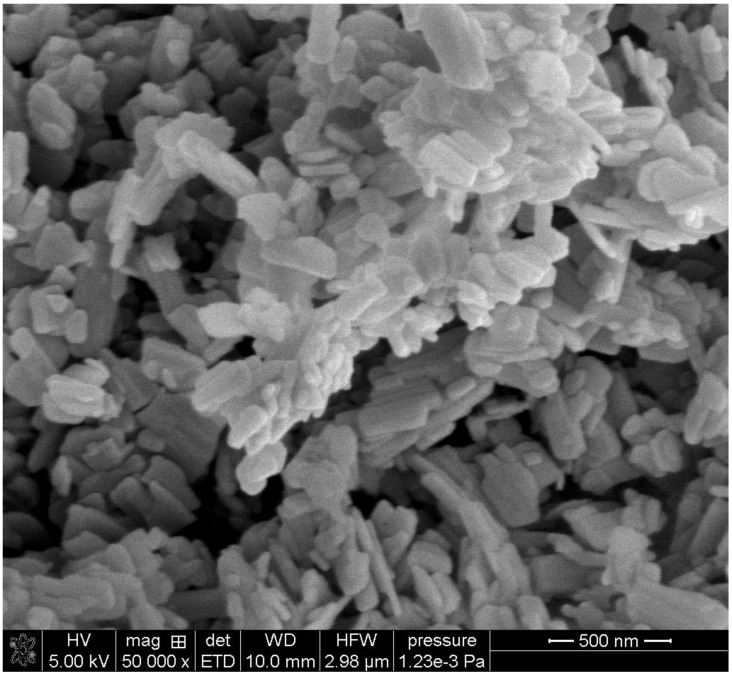
SEM image of brochantite.

**Figure 3:**
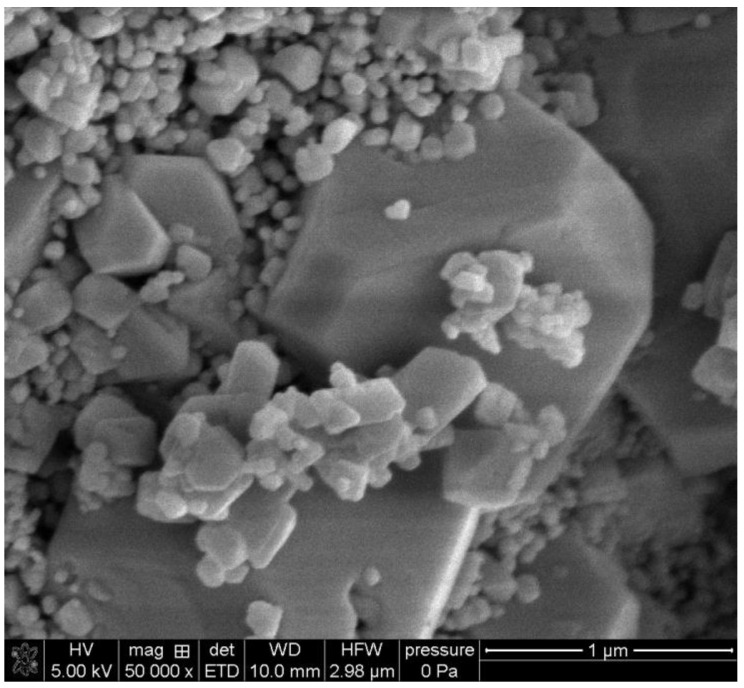
SEM image of paratacamite.

The SEM images of the initial particles are presented in Figure 2 and Figure 3.

Brochantite consists of irregularly shaped platelets about 50 nm thick, and the two other dimensions are on the order of 300 nm (Figure 2). Some platelets are interconnected. Paratacamite consists of octahedral particles of various sizes (Figure 3). The largest octahedrons are >1000 nm and the smallest are about 50 nm. Ul Haq et al. [15] obtained crystals of paratacamite of a similar shape, but their crystals were substantially larger than ours.

The specific surface areas of brochantite and paratacamite are reported in Table 1. The lower specific surface area of paratacamite compared to brochantite is consistent with the presence of large crystals in paratacamite (Figure 3) and their absence in brochantite (Figure 2).

**Figure 4:**
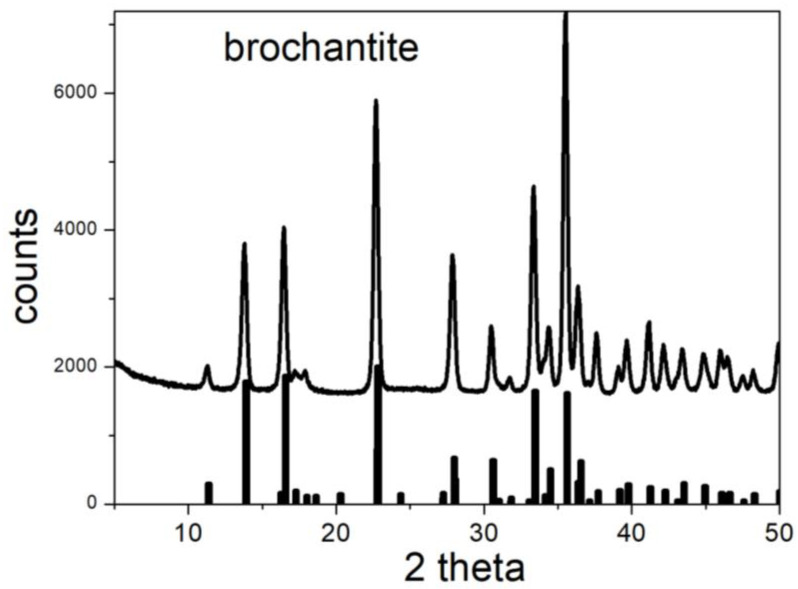
XRD pattern of brochantite. Line: experimental. Bars: from RRUFF repository.

**Figure 5:**
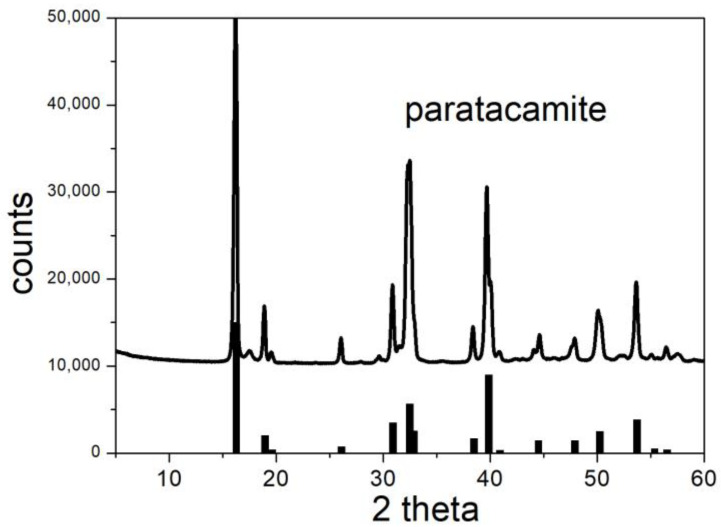
XRD pattern of paratacamite. Line: experimental. Bars: from RRUFF repository.

The structures of the initial materials were confirmed by XRD. Figure 4 and Figure 5 show that all of the major peaks of brochantite and paratacamite are present in the initial materials, and all reflexes in Figure 4 and Figure 5 can be assigned to brochantite and paratacamite, respectively.

**Figure 6:**
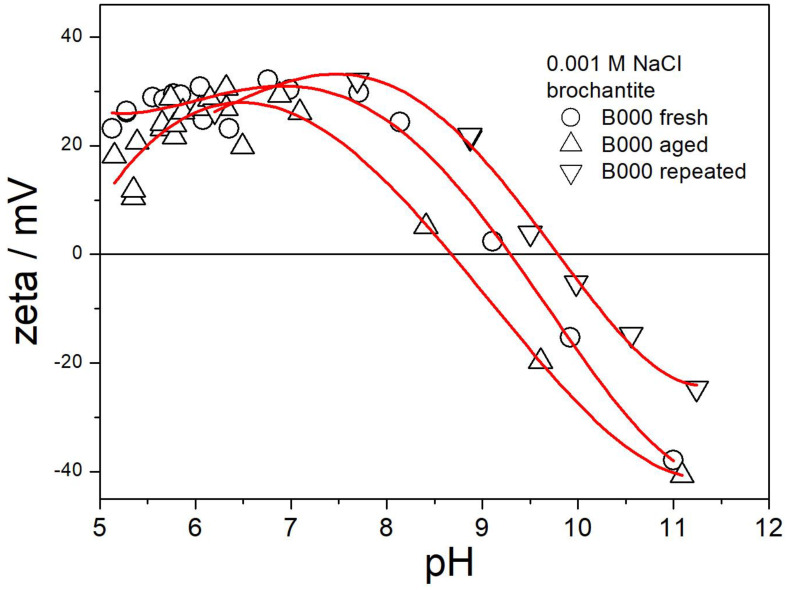
Electrokinetic curves of the original brochantite.

The electrokinetic curves of the original brochantite are reported in Figure 6.

The effect of pH on the ζ potential of brochantite is similar to a typical electrokinetic curve of metal oxides [16]. The ζ potential is positive at low pH and negative at high pH. The IEP (isoelectric point) is around pH 9.5. The ζ potential decreases with pH above the IEP. The positive ζ potential assumes its maximum value at a pH of about 7.5, and a further decrease in pH does not induce a further increase in the ζ potential, and even the ζ potential at pH 5 is lower than that at pH 6. Reaching a plateau in positive ζ potential 3–4 pH units below the IEP is common for metal oxides. Figure 6 illustrates the scatter of the results, which is typical for electrophoresis. The difference of ±5 mV between the ζ potentials obtained in two consecutive measurements with the same dispersion is commonplace. The reasons for such discrepancies are discussed in detail elsewhere [16].

### 3.2. Conversion of Brochantite by Ion Exchange

The presence of sulfate in the supernatant indicates the conversion of brochantite into another sparingly-soluble salt by ion exchange. The fractions of exchanged sulfate are summarized in Table 2.

The method used to determine the amount of sulfate in the supernatant in this study was not precise. Our primary goals were to:

- Recover the final product (sparingly soluble copper salt);
- Remove the water soluble compounds from the sparingly soluble copper salt.

These goals can be hardly reconciled with the precise determination of the fraction of exchanged sulfate. The apparent gain of sulfate above 100% might be due to co-precipitation of BaCO_3_ (we did not make any special attempts to exclude CO_2_ during the washing procedure) or co-precipitation of other Ba salts with BaSO_4_. These effects may be interesting by themselves, but their detailed study is beyond the scope of this work. The following conclusions can be drawn from Table 2.

- Anion exchange with chloride, bromide, and phthalate was successful;
- Anion exchange with nitrate, iodide, acetate, and rhodanide was not successful.

Table 2 confirms a few rules that correspond to the general laws governing the chemical kinetics:

- Longer exchange time (B008 vs. B007) leads to a higher degree of conversion;
- Higher temperature (B008 vs. B002) leads to a higher degree of conversion;
- Repetition of conversion (B001 vs. B002 and B014 vs. B004) leads to a higher degree of conversion.

However, the above rules have only a qualitative character, that is, they are not sufficient to predict the specific values of concentrations of reagents, equilibration time, and temperature, which are necessary to reach a substantial degree of conversion. For example, 1-day equilibration at room temperature was sufficient to almost completely convert brochantite into basic chloride, but it was not sufficient to convert brochantite into basic bromide.

The results from Table 2 were further confirmed by the XRD patterns.

**Figure 7:**
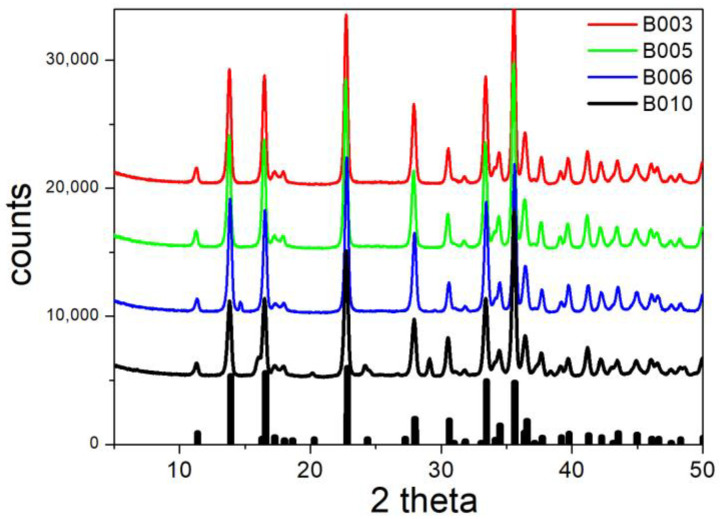
XRD patterns of B003, B005, B006 and B010. Lines: experimental. Bars: brochantite from RRUFF repository.

Figure 7 shows the XRD patterns of specimens in which the fraction of exchanged sulfate according to Table 2 was low. Not surprisingly, the XRD patterns of B003, B005, B006, and B010 are almost identical, and they are also very similar to the XRD pattern of the original brochantite (Figure 4). The only significant difference is a peak at 27.93 degrees in the XRD pattern of B010, which is absent in that of pure brochantite and in the other specimens presented in Figure 7. Apparently, this peak represents a compound containing copper and rhodanide. The highest peak in the XRD pattern of precipitated copper rhodanide is at 27.3 degrees. The amount of released sulfate (Table 2) was higher in B010 than in B003, B005, or B006, and this confirms a higher degree of conversion of brochantite into another copper salt. The failure of ion exchange with iodide is in line with our previous experiments [5], namely, alkalization of a solution containing Cu(II), sulfate, and iodide led to precipitation of brochantite rather than basic copper iodide.

Pereira et al. [17] reported the XRD patterns of basic copper acetate and nitrate and the main peaks were at 9.39 and 13 degrees, respectively. Such peaks are absent in the XRD patterns of B005 and B006, respectively.

**Figure 8:**
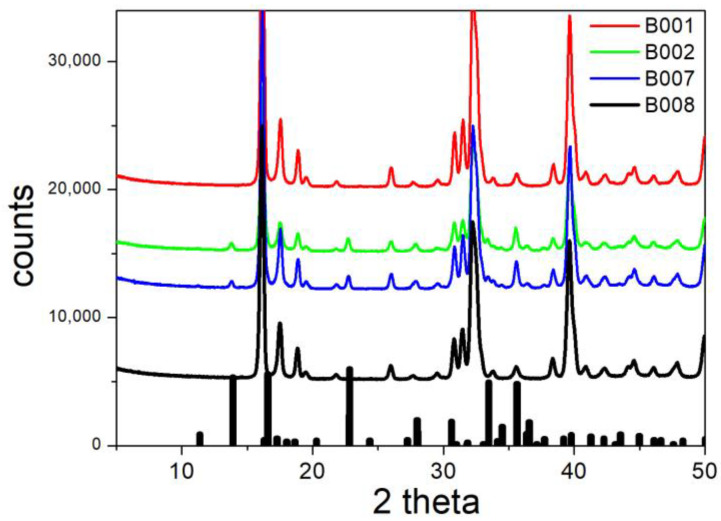
XRD patterns of B001, B002, B007, and B008. Lines: experimental. Bars: brochantite from RRUFF repository.

The XRD patterns of B001, B002, B007, and B008 (conversion of basic sulfate into chloride) are shown in Figure 8. The main brochantite peaks are absent for B001 and for B008, but small peaks at 13.91 and 22.82 degrees are visible for B002 and B007. These peaks indicate the incomplete conversion of brochantite, and they are in line with a low fraction of exchanged sulfate (Table 2) and with the aforementioned principles of chemical kinetics.

**Figure 9:**
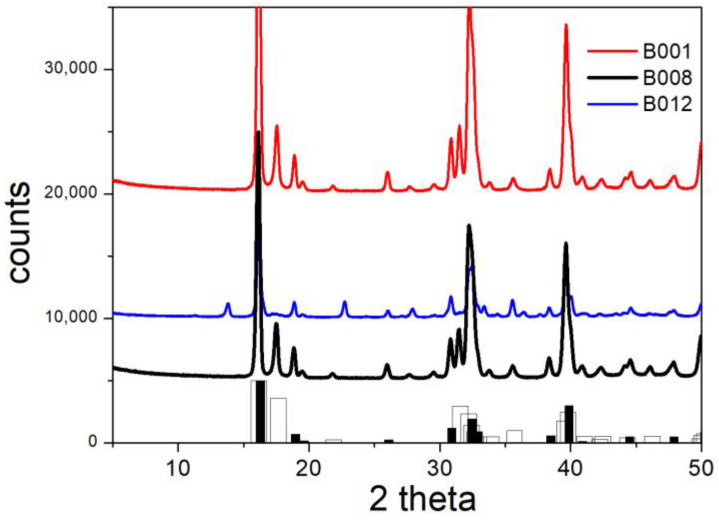
XRD patterns of B001, B008, and B012. Lines: experimental. Bars: atacamite (white) and paratacamite (black) from RRUFF repository.

The XRD patterns of B001, B008, and B012 (conversion of basic sulfate into chloride) are presented in Figure 9. The XRD pattern of B012 can be explained by the presence of paratacamite (no atacamite) with an admixture of brochantite (peaks at 13.91 and at 22.82 degrees, cf. Figure 8). By contrast, the XRD patterns of B001 and B008 (as well as those of B002 and B007, cf. Figure 8) indicate the presence of atacamite (a peak at 17.63 degrees, which is absent in the XRD pattern of paratacamite), with or without the admixture of brochantite.

While the presence or absence of brochantite in particular specimens can be explained by means of less or more complete ion exchange, the absence of atacamite in B012, in contrast with the presence of atacamite in B001, B002, B007, and B008, can be hardly explained, namely, the conditions of ion exchange in B012 and B008 were almost identical. Apparently, the reaction that occurred in specimens B001, B002, B007, B008, and B012 can be described as follows:

Cu_4_(OH)_6_SO_4_ + 2NaCl → 2Cu_2_Cl(OH)_3_ + Na_2_SO_4_ (3)

**Figure 10:**
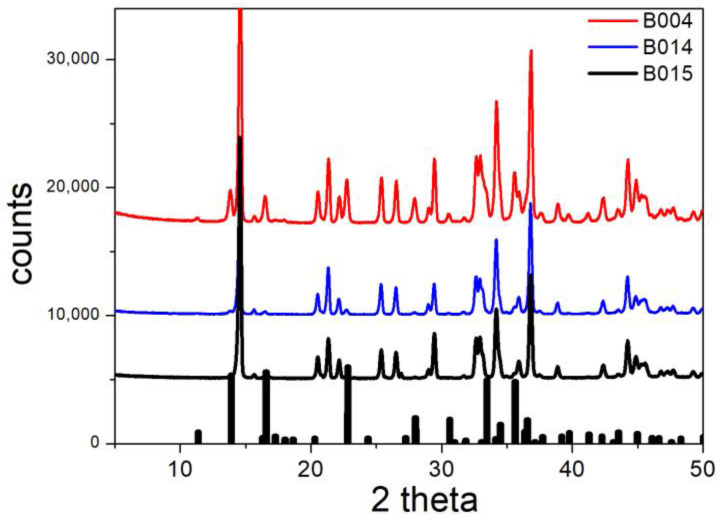
XRD patterns of B004, B014, and B015. Lines: experimental. Bars: brochantite from RRUFF repository.

The XRD patterns of B004, B014, and B015 (conversion of basic sulfate into bromide) are presented in Figure 10. The main brochantite peaks are absent for B014 and for B015 (except for a small peak at 22.82), but they are visible for B004. These peaks indicate the incomplete conversion of brochantite, and they are in line with a low fraction of exchanged sulfate in B004 (Table 2). The major peaks for B004, B014, and B015 match the peaks in the XRD pattern of monoclinic Cu_2_Br(OH)_3_ reported in [18]. Apparently, the following reaction occurred in B004, B014, and B015:

Cu_4_(OH)_6_SO_4_ + 2NaBr → 2Cu_2_Br(OH)_3_ + Na_2_SO_4_ (4)

The success of ion exchange with chloride (Figure 8 and Figure 9) and bromide (Figure 10) is somewhat surprising with respect to our previous experiments [5], namely, alkalization of a solution containing Cu(II), sulfate, and chloride (or bromide) led to precipitation of brochantite rather than basic copper chloride or bromide. Preferential precipitation of brochantite suggests that brochantite is less soluble than basic copper chloride or bromide, and Reactions (3) and (4) should rather go in the opposite direction.

**Figure 11:**
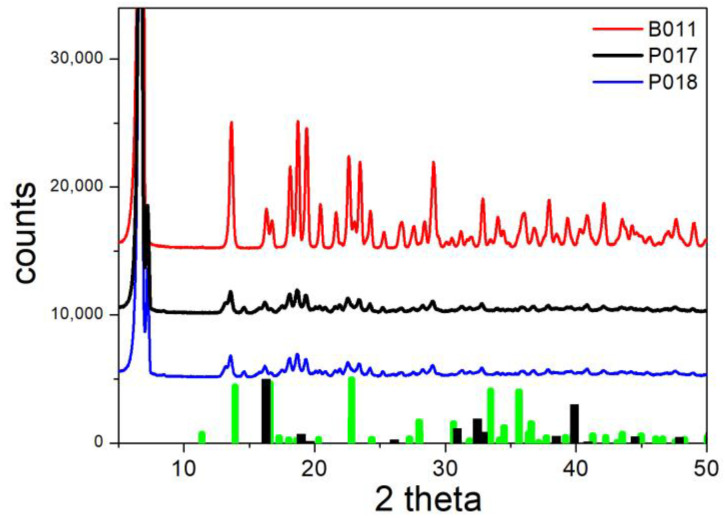
XRD patterns of B011, P017, and P018. Lines: experimental. Bars: brochantite (green) and paratacamite (black) from RRUFF repository.

The XRD patterns of B011, P017, and B018 (conversion of basic sulfate and basic chloride into phthalate) are shown in Figure 11. The main brochantite peaks are absent for B011 and the main paratacamite peaks are absent for P017 and P018. The absence of such peaks indicates the complete conversion of brochantite and paratacamite, and it is in line with a high fraction of exchanged sulfate (Table 2). The other peaks in Figure 11 are similar for B011, P017, and P018, and they match the peaks of copper phthalate reported in [19]. Meng et al. [19] attributed the peaks at 6.8, 13.7, 18.2, 18.7, and 19.9 degrees in their XRD pattern of copper phthalate to the (200), (-10-1), (020), (3-1-1), and (311) planes, respectively. The chemical identity of B018 was also confirmed by TGA, that is, the mass retained after heating at 500 °C under air flow roughly matched the fraction of CuO in CuC_8_H_4_O_4_. The nature of the ion exchange product was rather insensitive to the starting material (brochantite vs. paratacamite), except larger crystallites were obtained from brochantite (narrow peaks in the XRD pattern in Figure 11). Apparently, the following reactions occurred:

Cu_4_(OH)_6_SO_4_ + 4KHC_8_H_4_O_4_ → 4CuC_8_H_4_O_4_ + K_2_SO_4_ + 2KOH + 4H_2_O (5)

Cu_2_Cl(OH)_3_ + 2KHC_8_H_4_O_4_ → 2CuC_8_H_4_O_4_ + KCl + KOH + 2H_2_O (6)

The reaction environment was not alkalized as the result of Reactions (5) and (6) because the excess KHC_8_H_4_O_4_ used in these reactions acted as a pH buffer, and KOH was neutralized.

The results of our attempts to convert brochantite into other copper salts by ion exchange can be interpreted in terms of the solubility of copper salts. Theoretically, solubility products can be defined for each sparingly-soluble copper salt, but such solubility products are not useful in the systems of interest due to the plethora of reactions in solutions, including the equilibria between various copper complexes in solution involving various numbers of ligands, mixed ligands, and polynuclear complexes. The stability constants and even the nature of such complexes are not known. The adsorption of anions from solution on fine particles can also play some role. Therefore, we can hardly quantify our results, and they can only be presented in qualitative form. Apparently, copper basic acetate, nitrate, iodide, and rhodanide show higher solubilities than brochantite by several orders of magnitude, and this is why the attempts at ion exchange failed even with a substantial excess of these anions in solution. By contrast, copper basic chloride and bromide and copper phthalate show lower or similar solubilities as brochantite, and this is why the attempts at ion exchange were successful with sufficient excess of these anions in solution and sufficient equilibration times.

### 3.3. Conversion of Paratacamite into Brochantite by Ion Exchange

**Figure 12:**
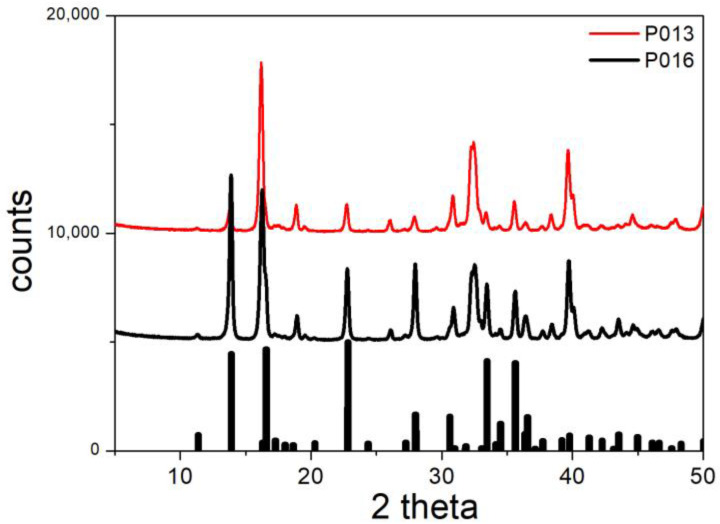
XRD patterns of P013 and P016. Lines: experimental. Bars: brochantite from RRUFF repository.

The XRD patterns of P013 and P016 (conversion of basic chloride into sulfate) are shown in Figure 12. Most of the main peaks in both XRD patterns can be explained by the presence of brochantite. Several major peaks of brochantite and paratacamite overlap, but the presence of trace amounts of paratacamite in P013 and P016 is confirmed by the paratacamite peaks at 26.11 and 32.46 degrees in Figure 12. Such peaks are not found for pure brochanite. Figure 12 confirms the partial conversion of paratacamite according to the following reaction:

2Cu_2_Cl(OH)_3_ + Na_2_SO_4_ → Cu_4_(OH)_6_SO_4_ + 2NaCl (7)

Reaction 7 is a reversed Reaction (3). In other words, the Reactions (3) and (7) are reversible when sufficient excess chloride (Reaction (3)) or sulfate (Reaction (7)) is used, and sufficient contact time is ensured to complete the reaction. The important difference is that only brochantite is produced in Reaction (7) (no other polymorphs), while Reaction (3) usually produces a mixture of paratacamite with atacamite. Our results are substantially different from those of Stanimirova et al. [7], who found that paratacamite and brochantite did not participate in ion exchange reactions. Apparently, the difference is due to the morphology of the particles. Fine particles with high specific surface area, undergo faster anion-exchange than larger particles with lower specific surface area.

### 3.4. Correlation Between the Exchanged Fraction and Specific Surface Area

In specimens B003, B005, B006, and B010, in which attempts at ion exchange failed, the specific surface area is 15.33–17.18 m^2^/g (Table 1), that is, it is similar to the specific surface area of the original brochantite. We emphasize that not all decimal digits in the specific surface area in Table 1 are significant. A relatively low specific surface area (15.33 m^2^/g) is observed in B010, which also showed a higher degree of conversion than B003, B005, and B006 (Table 2). By contrast, the specific surface area of brochantite substantially decreased in all specimens where ion exchange was successful. The specific surface areas are very different and they range from 1.26 to 5.31 m^2^/g in chloride-exchanged specimens, from 1.02 to 8.16 m^2^/g in bromide-exchanged specimens, and 1.73 m^2^/g in a phthalate-exchanged specimen. The specific surface area was usually low when the exchanged fraction was high. By contrast, specimen B004 with a low exchanged fraction had a relatively high specific surface area.

The sulfate-exchanged paratacamite (P013) had only a marginally lower specific surface area than the original paratacamite. The phthalate-exchanged paratacamites had specific surface areas of 1.15 and 1.23 m^2^/g, that is, 7 times lower than the original paratacamite. Their specific surface areas are similar to the aforementioned phthalate-exchanged brochantite.

### 3.5. ζ Potential and IEP

**Figure 13:**
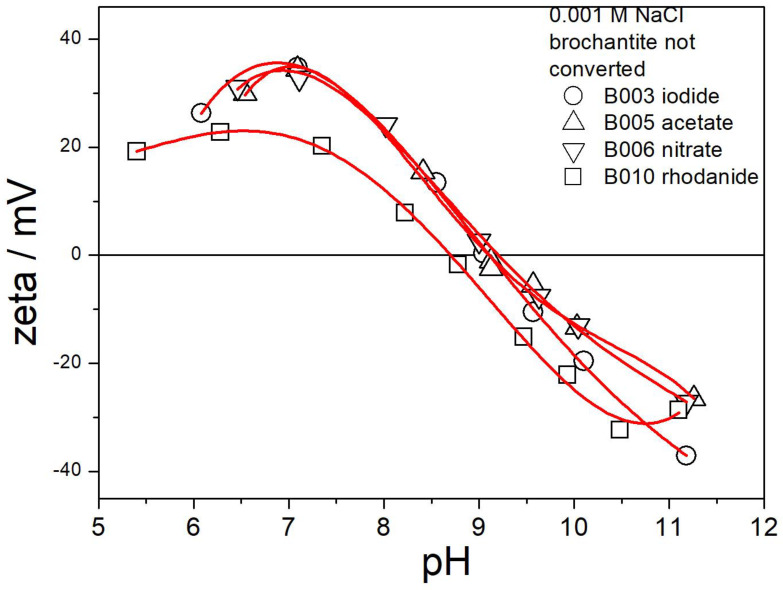
Electrokinetic curves of B003, B005, B006, and B010.

The electrokinetic curves of B003, B005, B006, and B010 are presented in Figure 13. Ion exchange in these specimens was not successful, so they consist chiefly of brochantite. Not surprisingly, the electrokinetic curves in Figure 13 are similar as those in Figure 6, including the numerical values of the ζ potential and the position of the IEP. In B010, the IEP is slightly shifted to low pH. This effect is due to the partial conversion of brochantite into a Cu-rhodanide compound, which caused an additional peak in the XRD pattern (Figure 7) and the release of 19% of sulfate in the ion exchange (Table 2). 

**Figure 14:**
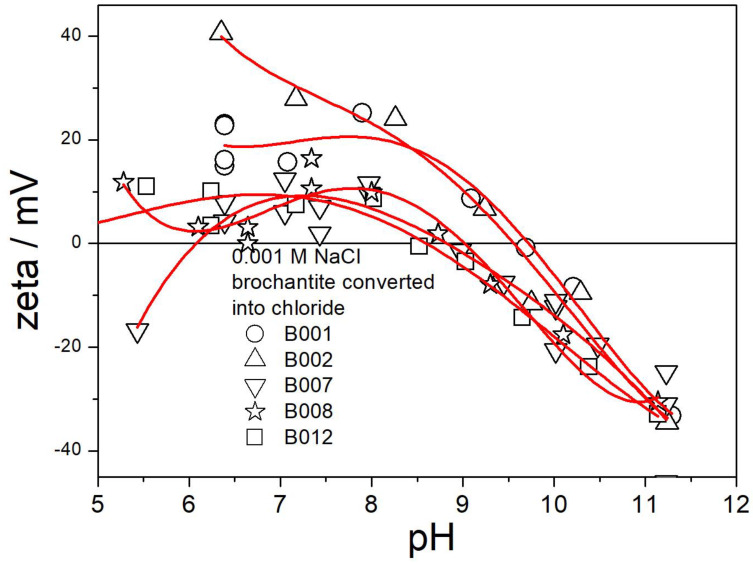
Electrokinetic curves of B001, B002, B007, B008, and B012.

The electrokinetic curves of B001, B002, B007, B008, and B012 (replacement of sulfate with chloride) are presented in Figure 14. Ion exchange in these specimens was successful, and they mainly consist of paratacamite (Figure 7 and Figure 8), with or without the admixture of atacamite and/or brochantite. The electrokinetic curves in Figure 14 are similar as those in Figure 6 and Figure 13 above pH 9. In B001 and B002 (ion-exchanged at room temperature) the IEP is at pH 9.5, as in the original brochantite, and the IEP was shifted to pH 9 in B007, B008, and B012. In B002 (low exchanged fraction), the entire electrokinetic curve is similar to the electrokinetic curves of brochantite presented in Figure 6 and Figure 13 over the entire pH range. The ζ potentials of B001, B007, B008, and B012 at pH < 9 are substantially lower than those of B002 and of not-exchanged brochantites (Figure 6 and Figure 13). The pH-dependent surface charging of basic salts depends on protonation and deprotonation of the surface oxygen atoms and on selective leaching of ions (here, chloride vs. hydroxide) from the surface layer [20]. Apparently, the replacement of sulfate with chloride makes a difference in selective leaching. Moreover, the leached-out sulfate (present as an impurity in basic copper chlorides obtained by ion exchange) may re-adsorb on the surface. Adsorption of sulfate anions is well known to induce negative surface charge of many materials, and this may be the case in the present study.

**Figure 15:**
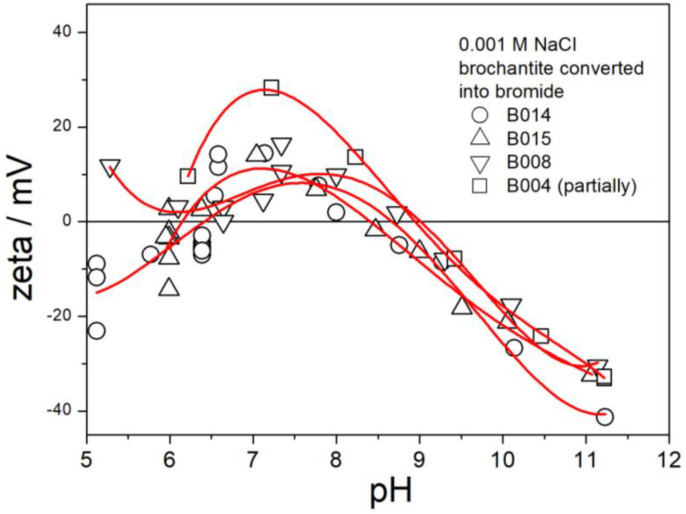
Electrokinetic curves of B004, B008, B014, and B015.

The electrokinetic curves of B004, B008, B014, and B015 (replacement of sulfate with bromide) are presented in Figure 15. Ion exchange in these specimens was successful except for B004, in which only about ½ of sulfate was exchanged (Table 2). The electrokinetic curves in Figure 15 are similar as those in Figure 6 and Figure 13 above pH 9, but the IEP is shifted to pH 9. The ζ potentials in Figure 15 at pH < 9 are substantially lower than those of not-exchanged brochantites (Figure 6 and Figure 13). Moreover, a few negative ζ potentials were observed at pH < 6.5. The positive branches of the electrokinetic curves in Figure 15 are similar to the positive branches of the electrokinetic curves in Figure 14. The similarity between the electrokinetic curves of chloride- and bromide-exchanged brochantites is not surprising in view of the chemical similarity between chlorides and bromides. A more substantial decrease in the positive ζ potentials in bromide-exchanged brochantites than in their chloride-exchanged counterparts may be due to higher exchanged fractions in the former.

**Figure 16:**
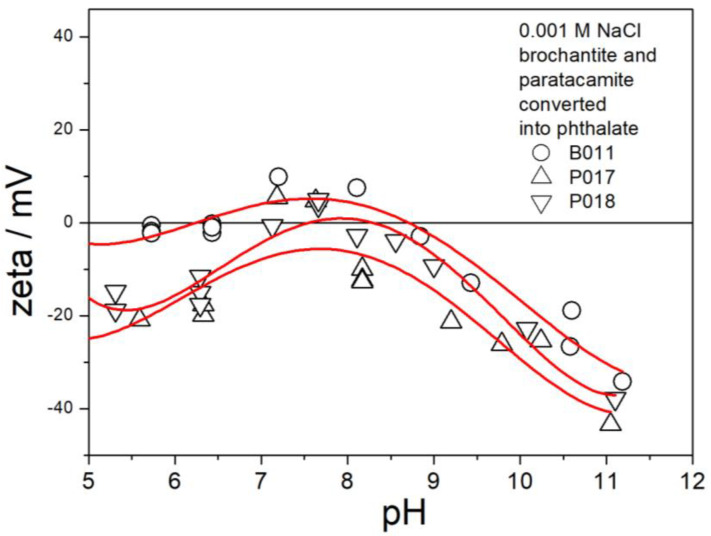
Electrokinetic curves of B011, P017, and P018.

The electrokinetic curves of B011, P017, and P018 (replacement of sulfate and chloride with phthalate) are presented in Figure 16. Ion exchange in these specimens was successful. The electrokinetic curves in Figure 16 are similar as those in Figure 6 and Figure 13 above pH 9, but the IEP is shifted to pH 8. Unlike in not-exchanged brochantites (Figure 6 and Figure 13) and in chloride- and bromide-exchanged brochantites (Figure 14 and Figure 15), the ζ potentials in Figure 16 below the IEP are only marginally higher than zero. The electrokinetic curves in Figure 16 do not follow the pattern observed in metal oxides and related materials (including basic copper salts), namely, the curves in Figure 16 are not symmetrical with respect to the IEP. CuC_8_H_4_O_4_ is not a basic salt, so it does not contain oxide or hydroxyl anions to be protonated or deprotonated. The negative surface charge of CuC_8_H_4_O_4_ may be due to the binding of hydroxyl ions from the solution by copper atoms in the copper–phthalate complex.

**Figure 17:**
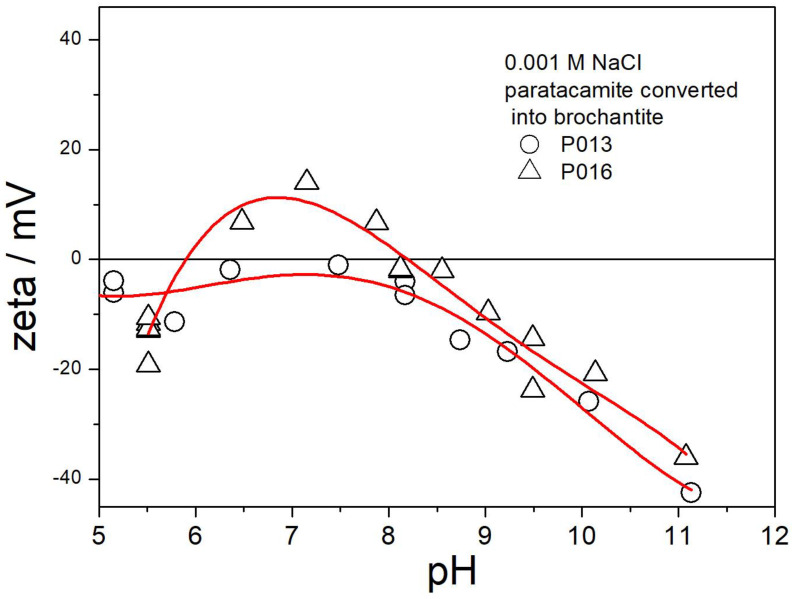
Electrokinetic curves of P013 and P016.

The electrokinetic curves of P013 and P016 (replacement of chloride with sulfate) are presented in Figure 17. Ion exchange in these specimens was successful. The electrokinetic curves in Figure 17 are similar as those in Figure 6 and Figure 13 above pH 9, but the IEP is shifted to pH 8. Unlike in not-exchanged brochantites (Figure 6 and Figure 13), the ζ potentials in Figure 17 below the IEP are positive only over a narrow pH range around pH 7. In other words, the brochantites obtained by anion exchange from paratacamite (P013 and P016) are very different in terms of their electrokinetic properties from the original brochantite obtained by precipitation (Figure 6). This example confirms that materials having similar chemical composition as detected by XRD and chemical analysis can substantially differ in their surface properties [20].

## 4. Conclusions

Fine particles of brochantite can be transformed into copper basic chloride, copper basic bromide, or copper phthalate by ion exchange when equilibrated with solutions containing corresponding anions. The materials obtained by ion exchange have substantially lower specific surface areas than the original brochantite. The original brochantite shows pH-dependent surface charging similar to that observed in metal oxides, and its IEP is at pH 9.5. Ion exchange leaves the basic branch of the electrokinetic curve unchanged, but the positive zeta potentials in ion-exchanged materials are decreased with respect to the original brochantite.

## Figures and Tables

**Table 1:** The specimens obtained by anion exchange.

Code	Brochantite [g]	Salt	Salt Mass [g]	Remarks	SSA [m^2^/g]
B000		None		original brochantite	16.26
P000		none		original paratacamite	8.70
B001	0.4999	NaCl	1.0001	then 1.0019 g of NaCl	2.52
B002	0.5036	NaCl	1.0011		5.31
B003	0.5026	KI	2.0012		17.18
B004	0.5002	NaBr	1.2047		7.44
B005	0.4999	NaCH_3_COO	1.2006		16.95
B006	0.5028	NaNO_3_	1.2014		17.07
B007	0.5091	NaCl	1.0057	1 day, 50 °C	4.17
B008	0.5060	NaCl	1.0007	50 °C	1.26
B009	0.5072	KHC_8_H_4_O_4_	2.0375		0.92
B010	0.5057	KSCN	2.0402		15.33
B011	0.5018	KHC_8_H_4_O_4_	2.0624		1.73
B012	0.5046	NaCl	1.0028	50 °C	1.91
P013	0.5013	Na_2_SO_4_·10H_2_O	2.0598		8.25
B014	0.5070	NaBr	1.2364	then 1.2469 g of NaBr, then 1.2092 g of NaBr	1.02
B015	0.5030	NaBr	3.1348	3 weeks	8.16
P016	0.5059	Na_2_SO_4_·10H_2_O	2.0029	then 2.0253 g of Na_2_SO_4_·10H_2_O, then 2.0074 g of Na_2_SO_4_·10H_2_O	1.55
P017	0.5011	KHC_8_H_4_O_4_	2.0032		1.23
P018	0.5033	KHC_8_H_4_O_4_	2.5135		1.15

**Table 2:** Fractions of exchanged sulfate (sulfate in supernatant: sulfate in the original brochantite).

Code	Anion	Fraction of Exchanged Sulfate
B001	Cl^−^	1.02
B002	Cl^−^	0.98
B003	I^−^	0.02
B004	Br^−^	0.52
B005	CH_3_COO^−^	0.05
B006	NO_3_^−^	0.09
B007	Cl^−^	0.91
B008	Cl^−^	1.19
B009	HC_8_H_4_O_4_^−^	1.24
B010	SCN^−^	0.19
B011	HC_8_H_4_O_4_^−^	1.33
B012	Cl^−^	1.12
B014	Br^−^	0.94
B015	Br^−^	1.12

## Data Availability

Data and materials are available from the authors upon request.
